# Aging affects high-density lipoprotein composition and function^☆^

**DOI:** 10.1016/j.bbalip.2013.06.004

**Published:** 2013-09

**Authors:** Michael Holzer, Markus Trieb, Viktoria Konya, Christian Wadsack, Akos Heinemann, Gunther Marsche

**Affiliations:** a Institute of Experimental and Clinical Pharmacology, Medical University of Graz, Austria; b Department of Obstetrics and Gynecology, Medical University of Graz, Austria

**Keywords:** Proteome, Cardiovascular disease, Paraoxonase, Anti-oxidative activity

## Abstract

Most coronary deaths occur in patients older than 65 years. Age associated alterations in the composition and function of high-density lipoproteins (HDL) may contribute to cardiovascular mortality. The effect of advanced age on the composition and function of HDL is not well understood.

HDL was isolated from healthy young and elderly subjects. HDL composition, cellular cholesterol efflux/uptake, anti-oxidant properties and paraoxonase activity were assessed. We observed a 3-fold increase of the acute phase protein serum amyloid A, an increased content of complement C3 and proteins involved in endopeptidase/protease inhibition in HDL of elderly subjects, whereas levels of apolipoprotein E were significantly decreased. HDL from elderly subjects contained less cholesterol but increased sphingomyelin. Most importantly, HDL from elderly subjects showed defective antioxidant properties, lower paraoxonase 1 activity and was more rapidly taken up by macrophages, whereas cholesterol efflux capability was not altered.

These findings suggest that aging alters HDL composition, resulting in functional impairment that may contribute to the onset/progression of cardiovascular disease.

## Highlights

• Aging remodels HDL protein and lipid composition.
• HDL from elderly subjects shows defective antioxidant properties and reduced paraoxonase 1 activity.
• Lipids from HDL of elderly subjects are more rapidly taken up by macrophages.

## Graphical abstract

**Figure:**
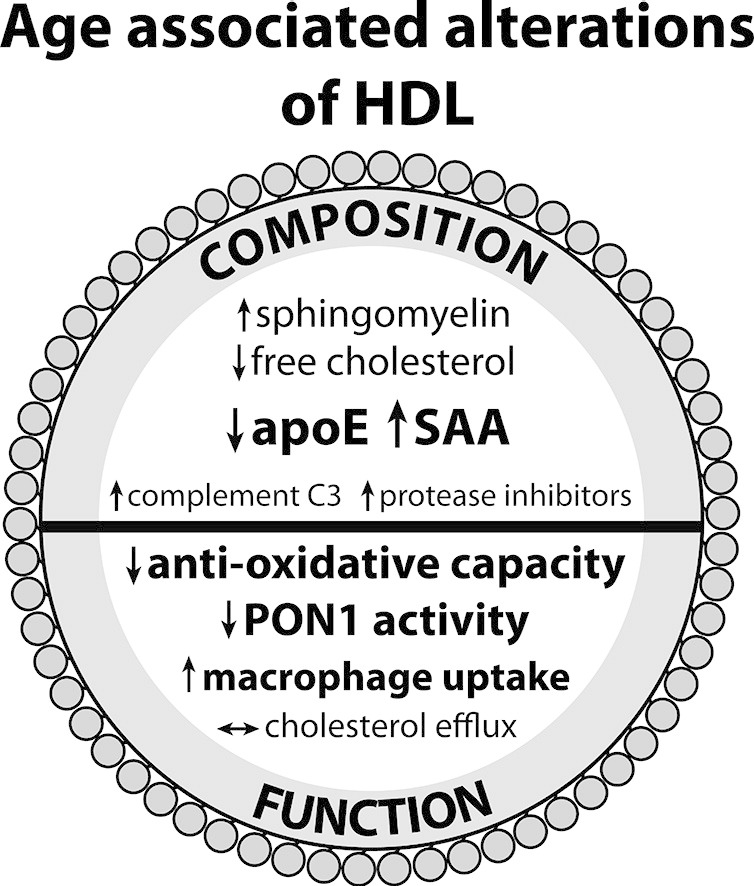

## 1 Introduction

Cardiovascular disease (CVD) is the leading cause of death worldwide. CVD rises dramatically with age and is of major concern in the increasing elderly population. Epidemiological studies have shown that high-density lipoprotein (HDL) cholesterol levels are inversely associated with risk for CVD [1]. The protective effect of HDL has been classically attributed to its ability to promote reverse cholesterol transport, a series of processes by which HDL is able to transport cholesterol from the periphery back to the liver for excretion [2]. Of particular interest, the ability of HDL to promote cholesterol efflux was found to be a better predictor for CVD than HDL-cholesterol [3]. In addition to its role in reverse cholesterol transport, HDL was found to inhibit low-density lipoprotein oxidation, to inhibit the secretion of pro-inflammatory mediators from macrophages, to reduce adhesion molecule expression on endothelial cells, to stimulate nitric oxide formation and to promote vasodilatation [4–8].

However, latest failures of HDL-cholesterol raising drugs and a recent study that showed no causal association between risk for myocardial infarction and genetically raised plasma HDL-cholesterol have called into question whether HDL-cholesterol is a suitable surrogate marker for HDL-related risk assessment [9,10].

Recent proteomic studies provided convincing evidence that inflammation alters the protein composition of HDL thereby generating dysfunctional or even pro-atherogenic forms of HDL [11] by enriching pro-inflammatory proteins such as serum amyloid A (SAA), apoC-III or complement component 3 [12–20]. In addition, inflammation leads to marked alterations in the lipid moiety of HDL, highlighted by a significant reduction in phospholipids [21–23].

These important studies linked compositional alterations of HDL with functional impairment of HDL, suggesting that even in the absence of low HDL-cholesterol levels, dysfunctional HDL may be causally involved in the development and progression of cardiovascular disease. Therefore, it is becoming increasingly apparent that direct measures of HDL function are needed rather than relying on surrogate markers such as the concentration of HDL-cholesterol.

Data on the effect of aging on HDL composition and function are limited. Associations of inflammation with age-related pathologies are documented; however, there is little information available how healthy aging impacts HDL composition and function. Initial studies reported that HDL from elderly subjects has a reduced potency to promote cholesterol efflux and to inhibit LDL oxidation [24,25]. In the present study, we assessed the impact of healthy aging on HDL composition and function.

## 2 Methods

### 2.1 Characteristics of study subjects and blood collection

All subjects were considered healthy and clinical characteristics are given in Table 1. Exclusion criteria included any history of cardiovascular disease, pregnancy, obesity, dyslipidemia, renal disease and diabetes. No subjects showed clinical signs of inflammation. Study subjects were free of lipid-lowering medication and anti-inflammatory drugs.

Blood was sampled from healthy subjects after obtaining written informed consent, according to a protocol approved by the Institutional Review Board of the Medical University of Graz (Nr.: 21-523 ex 09/10). Blood was collected in serum tubes (Greiner, Kremsmünster, Austria).

### 2.2 Isolation of HDL

Serum density was adjusted with potassium bromide (Sigma-Aldrich Corporation, Vienna, Austria) to 1.24 g/mL and a two-step density gradient was generated in centrifuge tubes (16 × 76 mm, Beckman Instruments) by layering the density-adjusted plasma (1.24 g/mL) underneath a NaCl-density solution (1.006 g/mL) as described [26,27]. Tubes were sealed and centrifuged at 90.000 rpm for 4 h in a 90 Ti fixed angle rotor (Beckman Instruments, Krefeld, Germany). After centrifugation, the HDL-containing band was collected, desalted via PD10 columns (GE Healthcare, Vienna, Austria) and immediately used for experiments or stored at − 70 °C.

### 2.3 Determination of plasma and HDL lipid composition

Levels of total cholesterol, non-esterified cholesterol, triglycerides, choline-containing phospholipids (DiaSys Diagnostic Systems GmbH, Holzheim, Germany) and sphingomyelin (Cayman Europe, Tallinn, Estonia) were measured enzymatically with commercially available kits. Sphingomyelin values were subtracted from total choline-containing phospholipids to quantify phosphatidylcholine. LDL cholesterol was calculated according to the Friedewald equation using HDL cholesterol values measured in the supernatant of the phosphotungstic acid/MgCl_2_ precipitation.

### 2.4 Biochemical quantification of HDL-associated proteins

ApoA-I, apoA-II, apoC-II, apoC-III and apoE (Greiner, Flacht, Germany) were determined by immunoturbidimetry. All lipoprotein analyses were performed on an Olympus AU640 analyzer (Olympus Diagnostika, Hamburg, Germany). Serum amyloid A (SAA) (Life Technologies, Vienna, Austria) and clusterin (BioVendor R&D, Candler, NC) were determined using enzyme-linked immunosorbent assays.

### 2.5 LC–MS/MS analysis

Proteomic profiling of HDL was performed as previously described [28]. HDL was digested with trypsin and the resulting peptides were separated by nano-HPLC. The sample was ionized in the nanospray source equipped with nanospray tips and analyzed in a LTQ-FT mass spectrometer (Thermo Scientific, Waltham, US). The standard deviation of spectral counts was below 10% between duplicates. Spectral counts were recorded and used for data analysis by searching the human SwissProt public database downloaded on May 4th 2011 with Spectrum Mill Rev. A.03.03.084 SR4 (Agilent Technologies, Vienna, Austria). Detailed search criteria were: trypsin; max. missed cleavage sites: 2; carbamidomethylation at cysteine as fixed modification; variable modification: oxidized methionine; precursor mass tolerance +/− 0.05 Da; product mass tolerance +/− 0.7 Da. Protein hits were subjected to automatic validation by Spectrum mill: for precursor charge of 2: score threshold 6.0, percent scored peak intensity (%SPI) threshold 60.0, Fwd-Rev score threshold 2.0 and rank 1-2 score threshold 2.0; for precursor charge of 1: score threshold 6.0, %SPI threshold 70.0, Fwd-Rev score threshold 2.0 and rank 1-2 score threshold 2.0; for precursor charge of 3: score threshold 8.0, %SPI threshold 70.0, Fwd-Rev score threshold 2.0 and rank 1–2 score threshold 2.0.

### 2.6 Advanced oxidation protein products (AOPP) assay

AOPPs have been determined as described previously with modifications [29,30]. Briefly, serum was depleted of apoB-containing lipoproteins with polyethylenglycol (PEG). 400 μL PEG-solution (20% PEG in 200 mmol/L glycine, pH = 7.4) was added per mL serum and incubated for 20 min at RT. Precipitate was pelleted (10,000 rpm, 30 min, 4 °C) and the supernatant used for AOPP detection. Subsequently, 10 μL apoB-depleted serum was mixed with 40 μL 0.2 mol/L citrate buffer and incubated for 2 min on a shaker. Afterwards, absorbance was measured at 340 nm. AOPP were calibrated with chloramine-T (linear within the range of 0 to 100 μmol/L) and were expressed as μmol/L of chloramine-T equivalents.

### 2.7 Arylesterase activity assay

Ca^2 +^-dependent arylesterase activity was determined with a photometric assay using phenylacetate as the substrate. HDL (0.5 μg protein) was added to 200 μL buffer containing 100 mmol/L Tris, 2 mmol/L CaCl_2_ (pH 8.0) and phenylacetate (1 mmol/L). The rate of hydrolysis of phenylacetate was monitored by the increase of absorbance at 270 nm and readings were taken every 30 s at room temperature to generate a kinetic plot. The slope from the kinetic chart was used to determine ΔAb_270nm_/min. Enzymatic activity was calculated with the Beer−Lambert Law from the molar extinction coefficient of 1310 mol^1^*L^− 1^*cm^− 1^ for phenylacetate.

### 2.8 Lp-PLA2 activity assay

Lp-PLA2 was measured using commercially available photometric assay (Cayman Europe, Talinn, Estonia).

### 2.9 LCAT, PLTP and CETP activity assay

LCAT was measured with a commercially available kit from Merck (Darmstadt, Germany). PLTP and CETP were measured with assay kits from Abnova (Eubio, Vienna, Austria).

### 2.10 Determination of the anti-oxidative capacity of HDL

The anti-oxidative activity of HDL was determined as previously described [31]. Briefly, dihydrorhodamine (DHR) was suspended in DMSO to a 50 mmol/L stock, which was diluted in HEPES (20 mmol/L HEPES, 150 mmol/L NaCl, pH 7.4) to a 50 μmol/L working reagent. 7.5 μg HDL protein was placed in a 384-well, 15 μL of DHR working reagent was added and the volume completed to 100 μL with HEPES buffer. The increase in fluorescence due to the oxidation of DHR was measured every 2 min for 1 h at 538 nm. The increase in fluorescence per minute was determined for samples containing only DHR and for samples containing DHR and individual HDL probes from study subjects.

### 2.11 Cholesterol efflux capability of HDL

Cholesterol efflux assay was performed as described previously [12]. Briefly, THP-1 macrophages, maintained in DMEM with 10% fetal bovine serum (FBS), were plated in 48-well plates and differentiated with 100 nmol/L phorbol 12-myristate 13-acetate (PMA). LDL was aggregated by vortexing at maximum speed for 2 min. Cells were lipid-loaded with 50 μg/mL aggregated LDL and labeled with [^3^H]cholesterol (1 μCi/mL) in medium containing 5% FBS for 24 h. After labeling, cells were washed and equilibrated in serum-free media containing 0.2% bovine serum albumin for 2 h. To determine [^3^H]cholesterol efflux, cells were incubated with 50 μg/mL HDL protein for 3 h at 37 °C. Supernatants were collected for liquid scintillation counting.

### 2.12 Uptake of HDL-lipids by THP-1 macrophage

HDL was labeled with 1,19-dioctadecyl-3,3,39,39-tetramethylindocarbocyanine perchlorate (DiI) as described previously [32]. Briefly, HDL was incubated with DiI (300 μg DiI per mg HDL protein) for 18 h at 37 °C and unreacted dye was removed by gel-filtration.

The human monocyte cell line THP-1 was seeded on 48-well plates in DMEM containing 10% FBS, 50 μg/mL aggregated LDL and 100 nmol/L PMA and grown overnight. Confluent cells were incubated with 25 μg/mL DiI-labeled HDL in serum-free DMEM, containing 2.5 mg/mL lipid-free BSA for 3 h at 37 °C. Cells were washed twice with PBS, detached with 100 μL accutase (PAA; Pasching, Austria) and transferred into flow cytometry tubes. Cells were fixed with formaldehyde and DiI-fluorescence was measured by flow cytometry.

### 2.13 Statistical analysis

Differences in plasma and HDL parameters between the two age groups were analyzed with the Student's *t*-test. Comparisons of multiple groups were done with One-Way ANOVA and Newman–Keuls post-hoc test. Correlations between compositional and functional data were determined with the use of Pearson product–moment estimates. Significance was accepted at *P < 0.05 and **P < 0.01. Statistical analyses were performed with SPSS Statistics Version 19.

## 3 Results

### 3.1 Aging is associated with altered composition of HDL

HDL was isolated from two groups of healthy subjects, with a mean age of 27.5 (median of age = 26.6, n = 26) and 68.0 (median of age = 67.2, n = 20), respectively. The two groups had no significant differences in serum lipid parameters including HDL-cholesterol levels (Table 1). Levels of C-reactive protein tended to increase, while SAA was significantly increased in serum of elderly subjects (Table 1).

Compositional analysis indicated that HDL from young subjects differed significantly in their protein and lipid composition (Table 2). HDL from elderly subjects had a reduced content of free and total cholesterol, whereas sphingomyelin content was increased (Table 2). To identify HDL-associated proteins, we performed immunoturbidimetry and enzyme-linked immunosorbent analysis (Table 2) for the major protein constituents and proteomics analysis for all protein constituents (Table 3). A significantly increased content of SAA and a reduced content of apoprotein E (apoE) was observed in HDL isolated from elderly subjects, whereas apoC-III and clusterin levels were not altered (Table 2). In addition, shotgun proteomics analysis revealed an increased content of complement C3 and proteins involved in endopeptidase/protease inhibition in elderly subjects, whereas the HDL-associated enzyme paraoxonase 1 was decreased (Table 3).

**Fig. 1:**
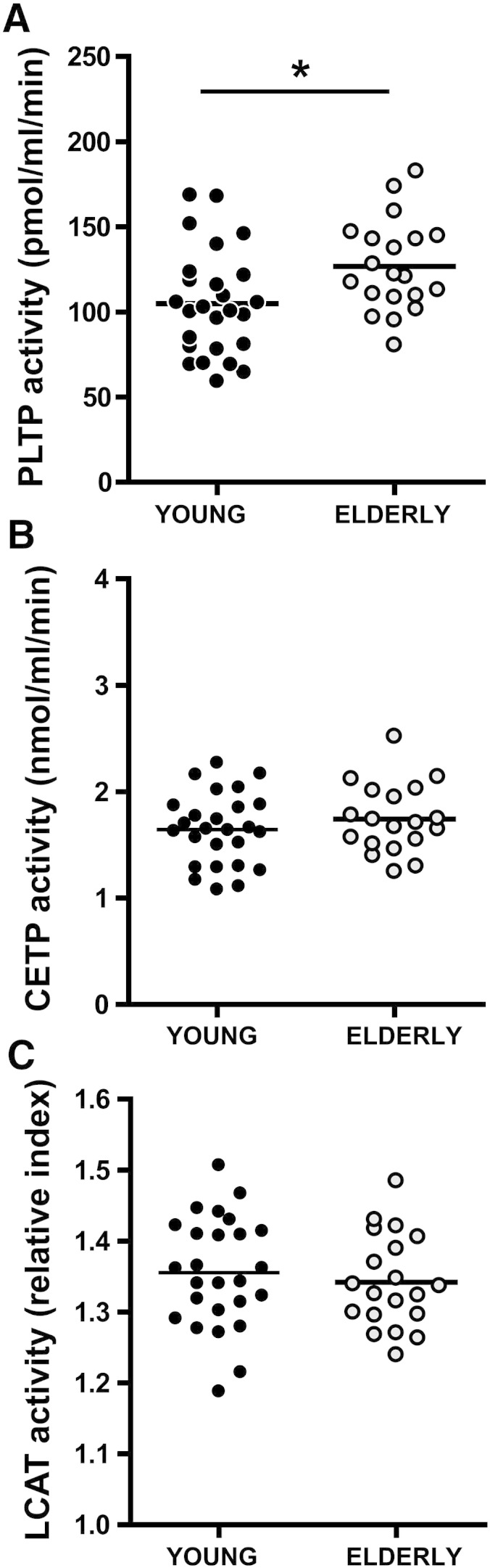
Activities of serum enzymes involved in HDL metabolism. Serum samples from two groups with a mean age of 27.5 (young, n = 26) and 68.0 (elderly, n = 20) were analyzed for the activities of (A) phospholipid transfer protein (PLTP), (B) cholesteryl ester transfer protein (CETP) and (C) lecithin-cholesteryl acyltransferase (LCAT). LCAT activity is presented as a relative index ranging from 1–2, where 1 indicated complete substrate conversion and 2 no substrate conversion. Significances were accepted at *P < 0.05.

Prompted by the observation that HDL composition is markedly altered, we examined important serum factors involved in the formation/maturation of HDL. We observed that the activity of phospholipid transfer protein (PLTP) was significantly higher in serum of elderly subjects (Fig. 1A), whereas activities of cholesteryl ester transfer protein (CETP) and lecithin-cholesteryl acyltransferase (LCAT) were unaltered (Fig. 1B, C).

### 3.2 Aging impairs HDL anti-oxidant capacity and paraoxonase activity

**Fig. 2:**
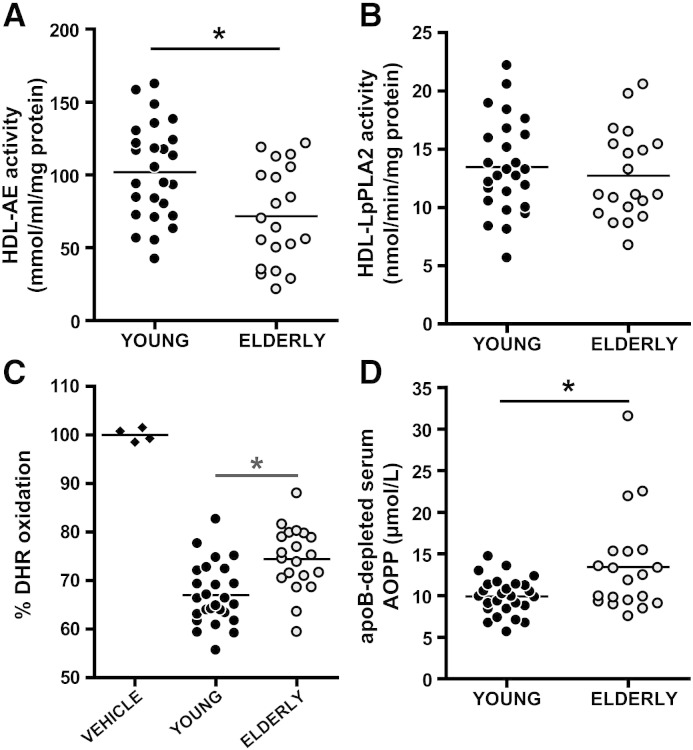
Anti-oxidative capacity of HDL is impaired by age. HDL from 26 young subjects and 20 elderly subjects was analyzed for its anti-oxidative potency. (A) Arylesterase activity of HDL-associated paraoxonase 1 (PON1) was measured by using phenylacetate as substrate. (B) Lipoprotein associated phospholipase A2 (Lp-PLA2) activity of HDL was measured using 2-thio PAF as substrate. The arylesterase and Lp-PLA2 activities of HDL were calculated from the slopes of the kinetic chart of three independent experiments. (C) Inhibitory activity of HDL on oxidation was measured by incubating HDL from young and elderly subjects with dihydrorhodamine (DHR). (D) Advanced oxidation protein products (AOPPs) were measured in apoB-depleted serum. Significances were accepted at *P < 0.05.

HDL has been reported to exhibit unique anti-oxidative activity based in part on observations that oxidative changes occur more slowly in LDL−HDL mixtures than in LDL alone [33]. We observed that the anti-oxidative activity of HDL to inhibit the oxidation of the fluorescent dye dihydrorhodamine from elderly subjects was significantly lower compared with HDL from younger subjects (Fig. 2C). Several proteins present on HDL are reported to metabolize lipid-peroxidation products of phospholipids, cholesteryl esters and triglycerides. Previous data suggested that HDL-associated paraoxonase 1 and/or lipoprotein associated phospholipase A2 (Lp-PLA2) contribute to the antiatherogenic activity of HDL [34–37]. We observed a significant reduction in paraoxonase 1 activity, whereas Lp-PLA2 activity was unaltered in HDL from elderly subjects (Fig. 2A, B). Interestingly, the impairment of HDL anti-oxidative properties was accompanied by increased formation of serum advanced oxidation protein products (AOPPs), an established marker of increased oxidative stress (Fig. 2D).

### 3.3 HDL-lipids from elderly subjects are taken up more rapidly by macrophages

**Fig. 3:**
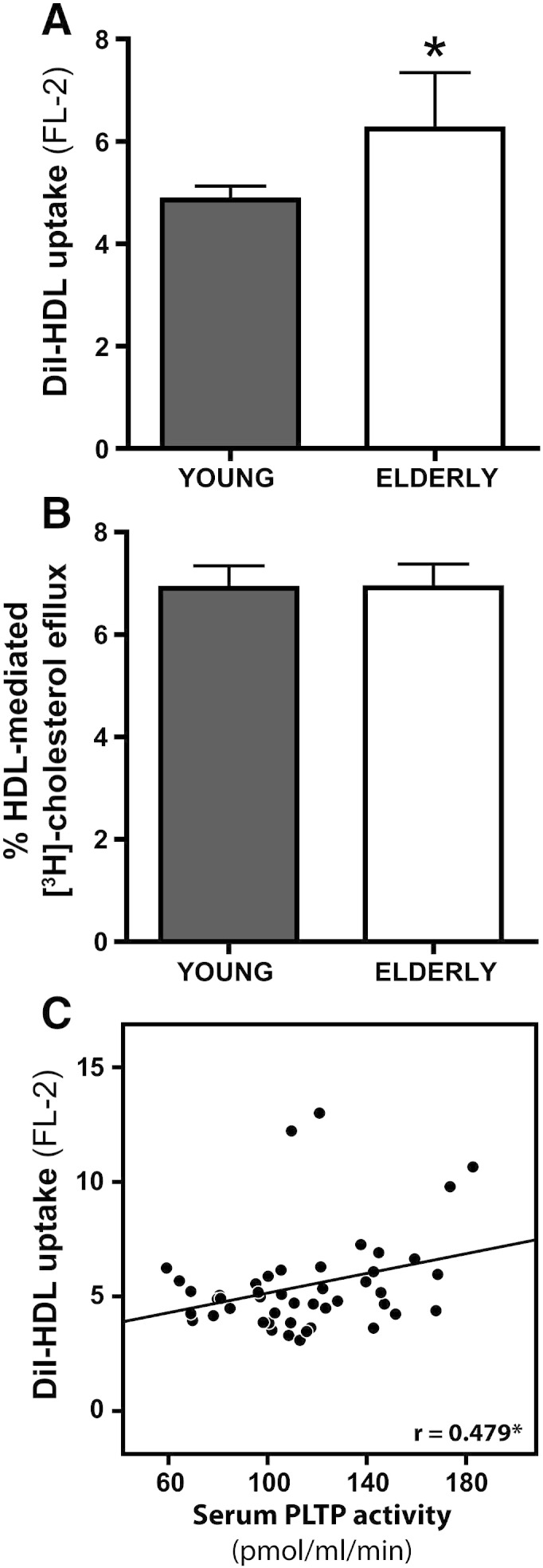
Increased macrophage uptake of lipids from HDL of elderly subjects. HDL isolated from 26 young subjects and 20 elderly subjects was examined for its ability to deliver lipids or mobilize lipids from macrophages. (A) THP-1 monocytes were differentiated into macrophages with 100 nM PMA and incubated with DiI-labeled HDL for 3 h. The uptake of the fluorescent lipophilic dye DiI was quantified by flow cytometry. Values shown represent means (± SD) of two individual experiments performed in duplicate. (B) To assess cholesterol efflux activity of HDL, [^3^H]cholesterol-labeled THP-1 macrophages were incubated with 50 μg/mL HDL for 3 h. Cholesterol efflux is expressed as radioactivity in the supernatant relative to total radioactivity (in supernatant and cells). Values shown represent means (± SD) of two individual experiments performed in duplicate. (C) Correlation between phospholipid transfer protein (PLTP) activity and DiI-HDL uptake of THP-1 macrophages. The Pearson correlation coefficients are noted. Significances were accepted at *P < 0.05.

A major determinant of plaque progression or regression rate is the balance between cholesterol uptake versus efflux pathways. To investigate the impact of age on the activity of HDL to remove cholesterol from macrophages, we analyzed the capacity of HDL from young and elderly subjects to mediate cholesterol uptake and cholesterol efflux from lipid-laden THP-1 macrophages. To assess whether HDL-lipid delivery is altered in elderly subjects, the HDL-lipid moiety was labeled with the fluorescent lipophilic dye DiI, which is a reliable surrogate to determine HDL-lipid delivery to cells [38]. We found that HDL from elderly subjects promoted lipid uptake more efficiently than HDL from young subjects (Fig. 3A). However, HDL from elderly subjects was as efficient in promoting cholesterol efflux from lipid-laden macrophages as HDL from young subjects (Fig. 3B). Interestingly, cellular DiI-HDL uptake significantly correlated with PLTP activity (Fig. 3C).

## 4 Discussion

The data presented here provide evidence that compositional changes in HDL from elderly subjects are linked to a loss of potential anti-atherogenic properties of HDL. These findings raise the possibility that dysfunctional HDL contributes to the high burden of cardiovascular disease in elderly subjects.

The compositional analysis clearly showed that the protein and lipid cargo of HDL from elderly subjects was profoundly altered, with markedly reduced levels of apoE, whereas several acute-phase proteins, such as SAA1, SAA2, α-1-antitrypsin, and α-1-acid-glycoprotein1 and proteins involved in complement activation, such as complement C3, were enriched. To validate proteomics analyses, we quantified the HDL-proteins apoA-I, apoA-II, apoC-II, apoC-III and apoE by immunoturbidimetry and SAA by enzyme-linked immunosorbent assays. HDL isolated from elderly subjects showed decreased free cholesterol content and was enriched in sphingomyelin. ApoE is produced by cholesterol-loaded macrophages, where it can promote cholesterol efflux during its secretion. ApoE is part of a gene cluster that is induced in macrophages by cholesterol-sensing nuclear receptors that protect against atherosclerosis in mice [39]. Interestingly, recent studies reported that chronic inflammation markedly remodels HDL composition associated with increased SAA and complement C3 content in HDL [11–13,16,40]. Complement C3 is produced by human monocyte-derived macrophages and contributes to innate immunity [41] and has been suggested to contribute to vascular disease [42]. A recent study demonstrated that SAA content in HDL from patients with chronic kidney disease is being responsible for pro-inflammatory effects of HDL. Incorporation of SAA into control HDL promoted cytokine production and adhesion molecule expression on monocytes and myeloid dendritic cells [14]. These findings are in line with the recent evidence indicating that SAA stimulates innate immune responses [43]. Of note, recent proteomics analysis of HDL also revealed that SAA is enriched in patients with acute coronary syndrome, psoriasis and rheumatoid arthritis indicating substitution of anti-inflammatory HDL with pro-inflammatory HDL during inflammation [11–13,16,18,40]. Therefore, increased SAA and complement C3 content of HDL observed in HDL of elderly subjects suggest that aging-induced alterations in the composition of HDL may play critical roles in the inflammatory response and lipid metabolism.

Most importantly, we observed that aging significantly alters the functionality of HDL. HDL-associated paraoxonase 1 was significantly lower in elderly subjects in agreement with previous studies [25,44]. In addition to reduced paraoxonase 1 activity, we observed increased systemic oxidative stress (measured as serum AOPP levels). This is in line with recent findings from genetic and biochemical studies showing that paraoxonase possesses pronounced systemic anti-oxidant activity in humans, with coincident links to a reduced risk of cardiovascular disease [45,46]. A recent clinical study provided evidence that the anti-oxidative activity of HDL may be of particular importance, since it was observed that the anti-oxidative capacity of HDL is significantly reduced in acute coronary syndrome, but not in stable coronary artery disease [37,47].

In regard to reverse cholesterol transport function of HDL, we observed that HDL-lipids are more rapidly taken up by macrophages from HDL of elderly subjects. Interestingly, serum PLTP activity was elevated in elderly subjects and correlated with macrophage uptake of HDL-lipids. This is in line with previous findings showing that elevated serum PLTP activity alters structure and composition of HDL resulting in increased cellular uptake of HDL-lipids [48,49]. Given that increased PLTP expression was reported in different pathologies associated with high risk of coronary heart disease, such as obesity, insulin resistance, and type I and II diabetes [50], accelerated uptake of HDL-derived lipids by macrophages might also contribute to cardiovascular risk in these subjects.

Of note, cholesterol efflux capacity of HDL did not significantly differ between the examined age groups. This is in line with the observation that phospholipid content of HDL, a critical component of the cholesterol acceptor capability of mature HDL, is not altered in elderly subjects [13,51]. It has to be mentioned that a previous study reported that cholesterol efflux capability of HDL from elderly subjects is reduced after long-term exposure of ^3^H-cholesterol labeled macrophages (24 h) with HDL [24]. Given that a significant fraction of effluxed ^3^H-cholesterol is taken up by macrophages within 24 h, an increased re-uptake of HDL-associated^3^H-cholesterol rather than a lower cholesterol mobilization capability of HDL may explain this apparently conflicting result.

### Study limitations

The sample size was small, retrospectively analyzed and from a single institution, and results are associative and do not prove a cause-and-effect relationship. However, our findings indicate that the composition of HDL is altered in elderly subjects and may help to develop rational therapeutic strategies that aim to increase HDL functionality. Consequently, the observed age associated alterations of HDL may contribute to the dramatically enhanced risk of cardiovascular disease in elderly subjects.

## Figures and Tables

**Table 1:** Clinical characteristics of study subjects.

	Young	Elderly
n	26	20
Age (yr)	26.6 (25.4–28.7)	67.2 (65.4–69.2)*
Male/female	13/13	9/11
CRP (mg/dL)	0.1 (0.0–0.2)	0.9 (0.5–2.9)
Total cholesterol (mg/dL)	174 (159–195)	225 (200–239)*
Triglycerides (mg/dL)	68 (54–102)	114 (89–130)
HDL-cholesterol (mg/dL)	55 (47–69)	56 (44–68)
LDL-cholesterol (mg/dL)	97 (85–113)	134 (122–160)*
SAA (mg/dL)	0.8 (0.4–1.5)	2.0 (0.8–7.9)**

Results are given as medians with interquartile range in brackets. Significances were accepted at the level of *P < 0.05.

**Table 2:** HDL composition.

μg/mg protein	Young	Elderly
*A) Lipids*		
Total cholesterol	245 ± 40	217 ± 35⁎
Cholesterylester	178 ± 29	164 ± 27
Free cholesterol	63 ± 14	52 ± 10⁎
Triglycerides	47 ± 26	54 ± 26
Phospholipids	450 ± 69	423 ± 58
Sphingomyelin	52 ± 10	61 ± 9⁎
*B) Proteins*		
apoA-I	535 ± 69	560 ± 48
apoA-II	149 ± 23	158 ± 21
apoC-II	5.5 ± 2.2	5.2 ± 2.6
apoC-III	23.5 ± 6.6	23.9 ± 8.0
apoE	17.0 ± 6.3	12.3 ± 4.7⁎
Clusterin	0.17 ± 0.08	0.16 ± 0.09
SAA	1.6 ± 1.2	6.8 ± 5.4⁎⁎

Results are given as mean ± SD. Apo, apoprotein; SAA, serum amyloid A.

⁎ P < 0.05.

⁎⁎ P < 0.01.

**Table 3:** Identification of proteins in HDL isolated from young and elderly healthy subjects.

Access. nr.	Protein name	Young	Elderly
		% HDL derived peptides	
P02647	Apo A-I	34.91	40.43
P02768	Albumin	13.79	11.39
P02649	Apo E	7.33	5.01
P05090	Apo D	6.18	5.01
P02654	Apo C-I	5.46	4.21
P02652	Apo A-II	4.17	3.99
P35542	SAA4	3.74	3.30
O95445	Apo M	3.30	3.19
P06727	Apo A-4	2.59	3.42
P27169	PON 1	2.59	2.05
P02775	Platelet basic protein	2.01	1.48
P08519	Lp(a)	2.01	1.25
P02656	Apo C-III	1.58	2.28
p02776	Platelet factor 4	1.58	1.59
P02735	SAA1	1.29	2.16
P04114	Apo B-100	1.29	1.14
P02655	Apo C-II	1.15	0.80
P00734	Prothrombin	1.01	1.14
O14791	Apo L1	0.86	0.68
P01009	α-1-Antitrypsin	0.72	1.03
Q13790	Apo F	0.72	0.68
P55056	Apo C-IV	0.43	0.46
Q15166	PON 3	0.43	0.46
PODJI9	SAA 2	0.29	0.57
P01834	IgK chain C region	0.14	0.11
P02766	Transthyretin	0.14	0.34
P02765	α-2-HS-GP	0.14	0.23
P02749	β-2-GP 1	0.14	0.11
POCG05	Igλ chain C region	0.00	0.11
P01857	Igγ chain C region	0.00	0.46
P02763	α-1-Acid GP 1	0.00	0.23
P02787	Serotransferrin	0.00	0.11
P01024	Complement C3	0.00	0.23
Q9BUN1	C1orf56	0.00	0.23

HDL was isolated from young (n = 26) and elderly (n = 20) healthy subjects by one-step-ultracentrifugation. The HDL proteome was analyzed of pooled fractions by an LC–MS/MS system and the data were analyzed by searching the human NCBI nonredundant public database with Mascot 2.2 (MatrixScience). Values shown represent non-quantitative estimates of the percentage of peptides of the total peptide count. The peptide count was 787 ± 129. Apo, apoprotein; PON, paraoxonase; SAA, serum amlyoid A; α-2-HS-GP, α-2-HS-glycoprotein; β-2-GP 1, β-2-HS-glycoprotein.
